# Relation of tricuspid annular displacement and tissue Doppler imaging velocities with duration of weaning in mechanically ventilated patients with acute pulmonary edema

**DOI:** 10.1186/1471-2261-10-20

**Published:** 2010-05-17

**Authors:** Vasilios E Papaioannou, Dimitrios A Stakos, Christos K Dragoumanis, Ioannis A Pneumatikos

**Affiliations:** 1 Department of Intensive Care Medicine, Democritus University of Thrace, Alexandroupolis University Hospital, Alexandroupolis, Greece; 2 Department of Cardiology, Democritus University of Thrace, Alexandroupolis University Hospital, Alexandroupolis, Greece

## Abstract

**Background:**

Liberation from the ventilator is a difficult task, whereas early echocardiographic indices of weaning readiness are still lacking. The aim of this study was to test whether tricuspid annular plane systolic excursion (TAPSE) and right ventricular (RV) systolic (Sm) and diastolic (Em & Am) tissue Doppler imaging (TDI) velocities are related with duration of weaning in mechanically ventilated patients with acute respiratory failure due to acute pulmonary edema (APE).

**Methods:**

Detailed quantification of left and right ventricular systolic and diastolic function was performed at admission to the Intensive Care Unit by Doppler echocardiography, in a cohort of 32 mechanically ventilated patients with APE. TAPSE and RV TDI velocities were compared between patients with and without prolonged weaning (≥ or < 7 days from the first weaning trial respectively), whereas their association with duration of ventilation and left ventricular (LV) echo-derived indices was tested with multivariate linear and logistic regression analysis.

**Results:**

Patients with prolonged weaning (n = 12) had decreased TAPSE (14.59 ± 1.56 vs 19.13 ± 2.59 mm), Sm (8.68 ± 0.94 vs 11.62 ± 1.77 cm/sec) and Em/Am ratio (0.98 ± 0.80 vs 2.62 ± 0.67, p <0.001 for all comparisons) and increased Ε/e' (11.31 ± 1.02 vs 8.98 ± 1.70, p <0.001) compared with subjects without prolonged weaning (n = 20). Logistic regression analysis revealed that TAPSE (R^2 ^= 0.53, beta slope = 0.76, p < 0.001), Sm (R^2 ^= 0.52, beta = 0.75, p < 0.001) and Em/Am (R^2 ^= 0.57, beta = 0.32, p < 0.001) can predict length of weaning ≥ 7 days. The above measures were also proven to correlate significantly with Ε/e' (r = -0.83 for TAPSE, r = -0.87 for Sm and r = -0.79 for Em/Am, p < 0.001 for all comparisons).

**Conclusions:**

We suggest that in mechanically ventilated patients with APE, low TAPSE and RV TDI velocities upon admission are associated with delayed liberation from mechanical ventilation, probably due to more severe LV heart failure.

## Background

Patients with acute cardiogenic pulmonary edema (APE) may some times experience severe acute respiratory failure that can lead to the application of controlled mechanical ventilation in the Intensive Care Unit (ICU). In these cases, discontinuation of ventilatory support is a challenging task and involves a careful weighting of the benefits of early extubation and the risks of premature spontaneous breathing trial (SBT). Both unnecessary delay and premature weaning may have adverse effects on patients' outcome, prolonging mechanical ventilation and length of ICU stay [1]. As a consequence, critical care physicians need accurate prediction tools for the early assessment of patients' ability to perform a successful SBT.

Doppler-echocardiography is increasingly used in the ICU for the estimation of both systolic and diastolic function of the left ventricle (LV) [2-5]. Recently, the combination of tissue Doppler imaging (TDI) and pulsed Doppler transmitral flow has been proven to reliably diagnose acute pulmonary edema as a cause of weaning failure in a mixed-population of critically ill patients without pre-existing heart disease [6]. However, prediction of successful liberation from the ventilator with echocardiography, before weaning is attempted has not been studied yet.

Therefore, in this observational study we tried to investigate a possible relation between different echo-derived indices obtained at admission and length of the weaning process, in a homogeneous group of patients with acute pulmonary edema, who were under controlled mechanical ventilation. The main question was if looking at the right ventricle (RV) through the estimation of simple and reproducible indices, such as tricuspid annular plane systolic excursion (TAPSE) and RV TDI velocities, could predict duration of weaning. For that reason, we also decided to correlate these measures with different echocardiographic indices of LV function, in order to estimate their accuracy in discriminating patients with more or less severe LV heart failure. In this case, changes of the right side of the heart could reliably reflect left sided findings, early in the course of liberating patients from the ventilator and therefore, identify a unique value of such analysis.

## Methods

### Setting and studying population

This study was performed in a mixed-12 bed ICU in the university hospital of Alexandroupolis, Greece, after approval by local scientific and ethics committee and after obtaining informed consent by patients' next of kin. A total of 32 patients admitted to the ICU from September 2008 to August 2009 were recruited, with a primary diagnosis of severe acute respiratory failure due to acute pulmonary edema. All patients survived and were discharged uneventfully from the ICU. APE was diagnosed in the Emergency Department [7]. Moreover, diagnosis was confirmed in the ICU by echocardiography, after excluding other causes of respiratory failure. According to recently published guidelines, subjects who fail at least three weaning attempts or require more than seven days after the first SBT, are considered to suffer a prolonged weaning [8,9]. Thus, we divided the whole study population in two groups depending on duration of the weaning process (< and ≥ 7 days). Patients with inappropriate acoustic windows, significant valvular pathologies and ventricular arrhythmia or atrial fibrillation were excluded from the study.

### Echocardiography

Echocardiographic examination was performed using available transthoracic ultrasound equipment (GE-vivid 3 with a phased-array transducer of 2.5 MHz; GE, Milwaukee, WI, USA) by the same operator (VP). Echocardiographic examination, including M-mode, two-dimensional (2D), pulsed and color Doppler measurements and TDI parameters were recorded at admission digitally for all patients and under controlled mechanical ventilation and analyzed off-line by an independent author specialized in echocardiography, who was blinded to study plan and clinical condition of the patients (DS). Measurements were done and evaluated according to recent guidelines [5].

Left ventricular ejection fraction (LVEF), was estimated after measuring left ventricular end-diastolic and end-systolic volumes using biplane Simpson's method from the apical two-and four-chamber views [10].

The maximal tricuspid regurgitation (TR) velocity was recorded by continuous-wave Doppler and pulmonary artery systolic pressure (PASP) was calculated using the modified Bernoulli equation, after estimating right atrial pressure (RAP) with a method developed by Lichtenstein [11-13]. Values of PASP ≥ 35 mmHg defined pulmonary hypertension.

Right ventricular fractional area change (RVFAC) was calculated as has been previously described [14]. TAPSE as a measure of RV base-to-apex shortening during systole was recorded on M-mode using the 2D four-chamber view. The cursor was placed to the junction of tricuspid valve plan with the free wall of the RV, whereas data were averaged over five beats as it has been recommended [15].

Pulsed Doppler tissue imaging techniques (TDI) were used for estimation of tricuspid annular systolic and diastolic velocities upon admission, after placing the cursor at the junction of the right ventricle free wall and the anterior leaflet of the tricuspid valve, using the 2D four-chamber view. The peak systolic (Sm), peak early diastolic (Em), and peak late diastolic (Am) annular velocities were calculated and averaged over three consecutive beats, as it has been previously described [16,17]. Furthermore, TDI of mitral annulus motion at its lateral aspect and calculation of Ε/e' was performed using PW Doppler [18,19]. Recordings were made at a sweep speed at 100 mm/sec.

### Statistical analysis

Two commercially available statistical programs (SPSS Software version 11.0, SPSS Inc, Chicago, Ill, USA and NCSS & PASS 2004, Statistical Systems, Kaysville, Utah, USA) were used. Results are expressed as mean ± standard deviation (SD) whereas a confidence level of p < 0.05 was taken as significant. Normal data distribution was estimated with Kolmogorov-Smirnov test. Pearson's correlations and multiple linear regression analysis were performed to the whole study group for calculating: 1. the relation between TAPSE and RV TDI velocities with Ε/e' and LVEF values at admission and 2. for estimating whether different echocardiographic indices were associated with duration of mechanical ventilation. Furthermore, differences of all echo-derived metrics were calculated with the Student's t-test, between subjects with and without prolonged weaning. In addition, we performed univariate and multivariate logistic regression analysis for identifying potential independent predictors of prolonged weaning and constructed receiver operating characteristics (ROC) curves for comparing accuracy of predictive indices and identifying cut-off values. For intraobserver and interobserver variability of LVEF, RVFAC, TAPSE, Sm, Em, Am and Ε/e'measurements, records were obtained in 10 random patients and both intra-and interclass correlation coefficients were computed.

## Results

### Patients' characteristics

The patients' characteristics and the echocardiographic data are depicted in Table 1. Patients did not differ in terms of age, body surface area (BSA), mean heart rate and arterial blood gases upon admission to the ICU. Half of the patients (n = 16, 50%) presented with pulmonary edema due to acute decompensation of chronic heart failure (CHF), that was attributed to lower respiratory track infection in 12 cases and unspecified reasons in the rest 4. The other 50% were diagnosed with de novo APE, due to acute coronary syndromes (5 cases), respiratory infections (with pre-existing and undiagnosed heart disease, 7 cases) and hematological or rheumatoid diseases (4 cases). None of the patients exhibited lactic acidosis upon admission to the ICU. From the whole study group, 22 patients were found to have pulmonary hypertension (including all patients with CHF) whether the remaining 10 had PASP < 35 mmHg. Furthermore, 12 subjects (10 suffering from CHF and 2 from rheumatoid diseases) experienced a prolonged weaning phase.

**Table 1:** Patients' characteristics for the whole studying population

Variables	Whole study group (n = 32)	Duration of weaning <7 days (n = 20)	Duration of weaning ≥ 7 days (n = 12)	p value
Gender (m/f)	26 (81%)/6 (19%)	16/4 (80%/20%)	10/2 (83%/17%)	NS
Age (years)	63.31 ± 5.23 (55-77)	63.85 ± 5.17	62.42 ± 5.45	NS
BSA (m^2^)	2 ± 0.29 (1.5-2.7)	2 ± 0.3	1.9 ± 0.2	NS
TAPSE (mm)	17.43 ± 3.16(12.2-24.2)	19.13 ± 2.59	14.59 ± 1.56	<0.001
Sm (cm/sec)	10.51 ± 2.08 (7.6-15.4)	11.62 ± 1.77	8.68 ± 0.94	<0.001
Em/Am	2 ± 1.07 (0.4-3.4)	2.62 ± 0.67	0.98 ± 0.80	<0.001
Ε/e'	9.85 ± 1.86 (7-13.2)	8.98 ± 1.70	11.31 ± 1.02	<0.001
LVEF (%)	40.13 ± 3.77 (32-46)	42.05 ± 3.05	36.92 ± 2.46	<0.001
RVFAC (%)	28.06 ± 5.26 (18-38)	30.95 ± 3.45	23.25 ± 4.11	<0.001
SBP (mm Hg)	129.7 ± 12.9 (99-152)	134.75 ± 8.50	121.50 ± 15.18	<0.05
PASP (mm Hg)	44.78 ± 10.38 (30-62)	38.65 ± 7.22	55 ± 5.59	<0.001
HR (beats/min)	85.75 ± 5.57 (73-97)	84.85 ± 5.72	87.25 ± 5.19	NS
Duration of controlled ventilation (days)	7 ± 3.18 (2-14)	4.6 ± 1.17	8.1 ± 3.2	<0.05

*NS: no significant, m/f: male/female*.

BSA: body surface area, TAPSE: tricuspid annular plane systolic excursion, Sm: systolic right ventricular (RV) tissue Doppler imaging (TDI) velocity, Em/Am: ratio of early (Em) vs late (Am) diastolic RV TDI velocities, Ε/e' lateral: ratio of maximal velocities of mitral PW Doppler E wave and TDI e' wave of the mitral annulus at the level of lateral wall, LVEF: left ventricular ejection fraction, RVFAC: right ventricular fractional area change, SBP: systolic blood pressure, PASP: pulmonary artery systolic pressure, HR: heart rate. Values are expressed as mean ± standard deviation (range).

All patients received intravenously nitrates and furosemide (10-20 mg/h), whereas 8 patients with CHF and prolonged weaning were given inotropes (levosimendan) according to published guidelines [20]. Time of intravenous administration was longer in patients with prolonged separation from the ventilator but did not reach statistical significance.

**Figure 1:**
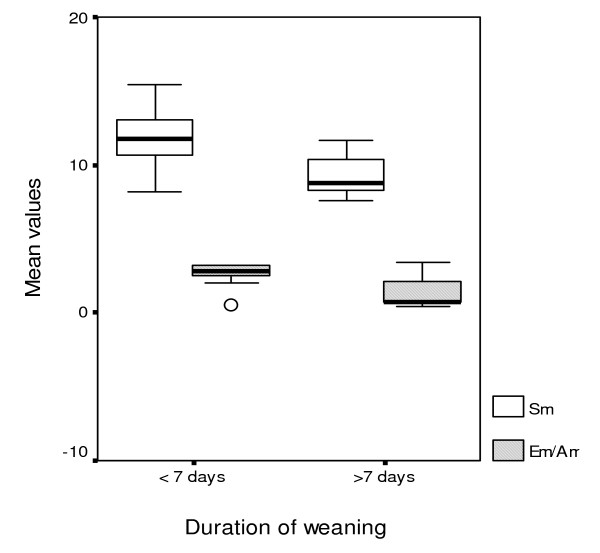
**Box plot illustrating Sm and Em/Am admission values versus the presence of prolonged weaning. **Box plot showing the significantly different distribution of Sm and Em/Am values at admission between patients with and without prolonged weaning (≥ or < 7 days after the first weaning trial, respectively).

**Figure 2:**
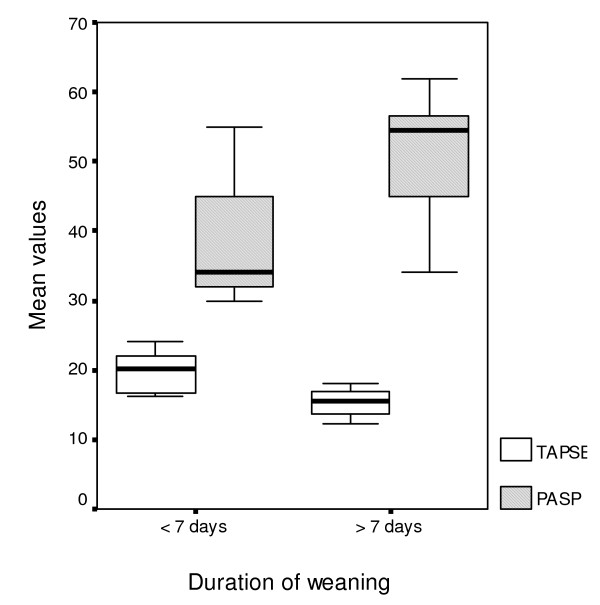
**Box plot illustrating TAPSE and PASP admission values versus the presence of prolonged weaning. **Box plot showing the significantly different distribution of TAPSE and pulmonary artery systolic pressure (PASP) values at admission between patients with and without prolonged weaning.

Patients with prolonged weaning (n = 12) were found to have significantly reduced values of all measured echo-derived indices, compared with those with shorter length of weaning (n = 20, Table 1, Figures 1 &2). Moreover, they exhibited significantly decreased systolic blood pressure (SBP) and increased PASP and Ε/e' values upon admission (Table 1).

### Correlations

TAPSE was found to be positively correlated with LVEF (r = 0.60, p < 0.05) and negatively correlated with Ε/e' lateral (r = -0.83, p < 0.001) and duration of mechanical ventilation (r = -0.74, p < 0.001) in the whole group of patients. Similarly, Sm was significantly correlated with Ε/e' lateral (r = -0.87, p < 0.001) and length of ventilatory support (r = -0.72, p < 0.001). Finally, Em/Am exhibited significant correlations with the same variables as Sm (r = -0.79, -0.67, p < 0.001 for both comparisons, respectively).

### Regression analysis

Linear regression analysis revealed significant associations between duration of ventilation and TAPSE [single R^2 ^= 0.55, multiple R = 0.74, beta slope = -0.89 with standard error (SE) = 0.14, p < 0.001], Sm (single R^2 ^= 0.52, multiple R = 0.72, beta = -0.57 with SE = 0.09, p < 0.001) and Em/Am (single R^2 ^= 0.45, multiple R = 0.67, beta = -0.27 with SE = 0.05, p < 0.001). The same pattern was observed between length of ventilation and Ε/e' lateral ratios but without statistical significance.

Logistic regression analysis revealed that TAPSE (R^2 ^= 0.53, multiple R = 0.73, beta = 0.76 with beta SE = 0.043, p < 0.001), LVEF (R^2 ^= 0.43, multiple R = 0.66, beta = 0.87 with beta SE = 0.03, p < 0.001), Sm (R^2 ^= 0.52, multiple R = 0.72, beta = 0.75 with beta SE = 0.03, p < 0.001), Em/Am (R^2 ^= 0.57, multiple R = 0.75, beta = 0.32 with beta SE = 0.05, p < 0.001) and RVFAC (R^2 ^= 0.52 multiple R = 0.72, beta slope = 0.74 with beta SE = 0.03, p < 0.001) can predict length of weaning ≥ 7 days. Multivariate analysis after adjustment of predictors found in univariate models for age, SBP, HR, BSA and duration of intravenous therapy concluded that they were independently associated with outcome of interest (p < 0.05). ROC curve analysis and calculation of areas under the curve (AUC) compared the accuracy of previously defined predictors and found particular cut-point values that were chosen to maximize the sum of sensitivity (positive test/condition present) and specificity (negative test/condition absent), (Table 2). TAPSE, LVEF and Sm had the higher AUC values whereas Sm with a cut-off value of 9.80 cm/sec predicted prolonged weaning with the higher combination of sensitivity (95%) and specificity (83%).

**Table 2:** ROC curve analysis of TAPSE, Sm, Em/Am, RVFAC and LVEF

Variable	Cut-off	AUC	AUC SE	95% CI	Sensitivity	Specificity
Sm	9.80	0.925	0.048	0.83-1.01	0.95	0.83
TAPSE	16.40	0.950	0.035	0.88-1.01	0.85	0.75
Em/Am	0.80	0.892	0.066	0.76-1.01	0.95	0.66
RVFAC	25%	0.887	0.065	0.76-1.01	0.95	0.75
LVEF	39%	0.927	0.050	0.83-1.02	0.84	0.80

Receiver operating characteristics (ROC) curves of TAPSE, Sm, Em/Am, RVFAC and LVEF in prediction of duration of weaning process. The areas under the curve (AUC), along with their standard error (SE) and 95% confidence intervals (CI), as well as the cut-off values of studying variables are given with their respective sensitivity and specificity.

### Reproducibility of measured variables

Correlation coefficients estimating intraobserver reproducibility of LVEF, RVFAC, TAPSE, Sm, Am and Ε/e' were: r = 0.73, 0.68, 0.84, 0.86, 0.84 and 0.82 whereas coefficients for interobserver variability of the same measurements were: r = 0.69, 0.71, 0.82, 0.75, 0.76 and 0.79 respectively, (p < 0.05 for all comparisons).

## Discussion

In this study we demonstrated that TAPSE and RV TDI velocities obtained by 2D echocardiography were significantly correlated with Ε/e', LVEF and length of ventilatory support in a cohort of mechanically ventilated patients with acute pulmonary edema. Furthermore, these indices were proven to discriminate successfully patients with different duration of weaning.

Liberation from mechanical ventilation can impose a significant load upon the cardiovascular system, especially in patients with heart disease. The shift from positive to negative intrathoracic pressure can augment venous return and central blood volume, increasing left ventricular preload and afterload. Moreover, high levels of catecholamines may reduce supply of oxygen to the heart due to tachycardia and decrease cardiac output due to increased pulmonary and vascular resistance [21-23].

Different studies in mechanically ventilated patients have confirmed that increased pulmonary artery systolic and occlusion pressure (PAOP) during the weaning trial are associated with weaning failure [21,24] whereas only one study using echocardiography investigated the relation between the echo-derived index Ε/e' and PAOP during liberation from the ventilator, in patients without pre-existing heart diseases [6]. However, baseline filling pressures have not been proven to successfully predict weaning outcome [21,25], whereas early assessment of weaning readiness with echocardiography during the course of mechanical ventilatory support has not been studied yet. In a recent study, high levels of B-type natriuretic peptide (BNP) just before the performance of weaning trial in a mixed population of patients were identified as independent risk factors for weaning failure [26].

In our study, we decided to investigate the prognostic impact of different RV echo-derived indices upon duration of weaning, which have been proven to be easily reproduced and obtained [15-17], without suffering technical limitations of LV derived measures, such as LVEF [10]. We also used the Ε/e' lateral ratios as an indirect measure of LV filling pressures, since this index is highly reproducible [18,19], whereas a threshold value more than 9.5 has been proven to predict PAOP values higher than 18 mmHg [19]. We finally hypothesized that looking early at the right side of the heart might be more easy and accurate than estimating different load dependent variables of the left side, in case that significant correlations were found between each other.

Numerous studies have documented TAPSE is linearly related to right ejection fraction in different clinical scenarios such as ischemia, congestive heart failure and pulmonary hypertension, whereas its measurement has been proven to be highly reproducible and easy to obtain [15,27-30]. Its value in the ICU setting was explored in a recent investigation by Lamia et al, who studied for the first time the relation of TAPSE with both LV and RV function in critically ill patients with cardiovascular and respiratory failure and found a significant correlation between TAPSE and LVEF [30].

Tissue Doppler imaging (TDI) techniques measure velocities of cardiac tissue and reflect directly myocardial function. Meluzin et al has found that systolic velocities Sm correlate with RVFAC and prognosis of patients with LV heart failure [31,32]. Dokainish has shown that Sm detected mild degrees of RV dysfunction not yet apparent from visual assessment and found that Sm was an independent predictor of cardiac death or rehospitalization in patients with LV heart failure [33].

The direct correlations that were found in our study between low TAPSE and RV TDI velocities with LVEF reflect systolic ventricular interdependence, while the inverse correlations between the RV function parameters and Ε/e' indicate that LV filling pressures comprise an important part of RV afterload in the context of acute pulmonary edema [34]. Consistent with prior studies, the measurements of TAPSE Sm, Em/Am and Ε/e' were highly reproducible with acceptable interobserver and intraobserver agreement. On the contrary, RVFAC and LVEF measurements' reproducibility was moderate, since the variability of these metrics reflects inaccurate endocardial visualization and dependence upon mathematical assessment [14,29].

The fact that ten patients from the whole study group did not have pulmonary hypertension was peculiar; however, this cohort consisted of subjects without a history of CHF. In addition, the 12 patients who experienced prolonged weaning had increased PASP and included mostly patients with decompensated chronic heart disease. Moreover, technical reasons concerning PASP estimation cannot be excluded since, different levels of tidal volume (TV) and positive end-expiratory pressure (PEEP) may alter inferior vena cava (IVC) diameter. Right atrial pressure depends not only on RV preload but also on pleural pressures transmitted to the right atrium, leading to differences between intravascular and transmural pressures under mechanical ventilation. For that reason, measurement of distensibility index (dIVC) of IVC has been proposed for RAP estimation in ventilated patients [35]. However, we did not apply this method in our study. Finally, different effects and time onset of therapy (both ventilation and drug administration) before performance of echo measurements could be another reason of low PASP values in some of our patients.

Since PAOP estimation loses prognostic value with time from initial diagnosis due to rapid change in response to treatment, Ε/e' ratio could not accurately discriminate patients according to different length of weaning. On the contrary, significantly increased AUC values of TAPSE, RV TDI velocities, RVFAC and LVEF possibly reflect a biventricular failure with a more severe effect of increased LV filling pressures upon RV performance, in the subgroup of patients with prolonged separation from the ventilator.

The above associations between TAPSE and RV TDI velocities with length of weaning may have limited validity due to the study design. First, the combination of a small sample size with multiple comparisons between different subgroups might have predisposed to both false positive and false negative results. Second, we did not use pulmonary artery catheter (PAC) for estimating PASP, RV and LV function. As it has been suggested, assessment of RV and LV filling pressures with PAC seems to be inaccurate, especially during mechanical ventilation [36].

Third, the associations being found do not imply causality. More studies with bigger sample size are needed to confirm possible significance of our results, whereas other echocardiographic indices could also be tested or even compared with those used in our study for early prediction of weaning outcome.

## Conclusions

In conclusion, we suggest that reduced TAPSE and tricuspid annular TDI systolic and diastolic velocities are associated with increased duration of weaning in critically ill patients with pulmonary edema, reflecting a more severe LV heart failure. These measurements are fast and easily reproducible, can accurately estimate LV performance even by less experienced operators in the ICU setting, whereas their adoption for weaning outcome assessment in different groups of patients merits further investigation. A comparison with other indices of cardiac function, like BNP serum levels and an associated change with different treatment protocols could also be investigated in future studies.

## Abbreviations

**APE**: acute pulmonary edema; **BNP**: B-type natriuretic peptide; **BSA**: body surface area; **CHF**: chronic heart failure; **CW**: continuous wave; **ICU**: Intensive Care Unit; **IVC**: inferior vena cava; **LVEF**: left ventricular ejection fraction; **LV**: left ventricle; **PAOP**: pulmonary artery occlusion pressure; **PASP**: pulmonary artery systolic pressure; **PEEP**: positive-end expiratory pressure; **PW**: pulsed-wave; **RAP**: right atrial pressure; **RV**: right ventricle; **RVFAC**: right ventricular fractional area change; **SBP**: systolic blood pressure; **SBT**: spontaneous breathing trial; **TAPSE**: tricuspid annular plane systolic excursion; **TDI**: tissue Doppler imaging; **TR**: tricuspid regurgitation; **TV**: tidal volume.

## Competing interests

The authors declare that they have no competing interests.

## Authors' contributions

All authors have read and approved the final manuscript. **VP**: Was the principal investigator who designed the study, made echo calculations, statistical analyses and wrote the manuscript. **DS**: Was associate investigator who also performed echo calculations and chequed the first author's echo-derived measurements. **CD**: Helped with data gathering and statistical analysis. **IP**: He reviewed the manuscript and helped in final submission format.

## Pre-publication history

The pre-publication history for this paper can be accessed here:

http://www.biomedcentral.com/1471-2261/10/20/prepub
